# Supplementary material for: Early proteostasis of caveolins synchronizes trafficking, degradation, and oligomerization to prevent toxic aggregation
Source: J Cell Biol. 2023 Aug 1;222(9):e202204020. doi: 10.1083/jcb.202204020 (PMC10394380; doi:10.1083/jcb.202204020)
Supplement: Table S1 — shows the key resources used in this study. [file JCB_202204020_TableS1.docx]

**Table S1. Key Resources Table.**

| **REAGENT or RESOURCE** | **SOURCE or REFERENCE** | **IDENTIFIER** |
| --- | --- | --- |
| **Deposited Data** | | |
| human CAV1 cryoEM structure | Porta et al., 2022, Sci. Adv. | PDB ID XXXX and EMDB ID XXXX |
| human CAV1 sequence | Uniprot | Q03135 (isoform α)  Q03135-2 (isoform β) |
| human CAV2 sequence | Uniprot | P51636 |
| human CAV3 sequence | Uniprot | P56539 |
| **Software** | | |
| Pymol | Schrodinger, USA. | <https://pymol.org/2/> |
| AlphaFold2 and AlphaFold2-Multimer | (Evans et al., 2022; Jumper et al., 2021) | <https://github.com/deepmind/alphafold> |
| ColabFold | (Mirdita et al., 2021) | <https://colab.research.google.com/github/sokrypton/ColabFold/blob/main/AlphaFold2.ipynb?authuser=3> |
| Multalin (sequence alignment) | (Corpet, 1988) | <http://multalin.toulouse.inra.fr/multalin/multalin.html> |
| ESPRipt (alignment coloring) | (Robert and Gouet, 2014) | <https://espript.ibcp.fr/ESPript> |
| Protein-Sol Patches (surface hydrophobicity) | (Hebditch and Warwicker, 2019) | <https://protein-sol.manchester.ac.uk/patches> |
