# Supplementary figures and images for: Early proteostasis of caveolins synchronizes trafficking, degradation, and oligomerization to prevent toxic aggregation
Source: J Cell Biol. 2023 Aug 1;222(9):e202204020. doi: 10.1083/jcb.202204020 (PMC10394380; doi:10.1083/jcb.202204020)

Source Data Fig. 2B

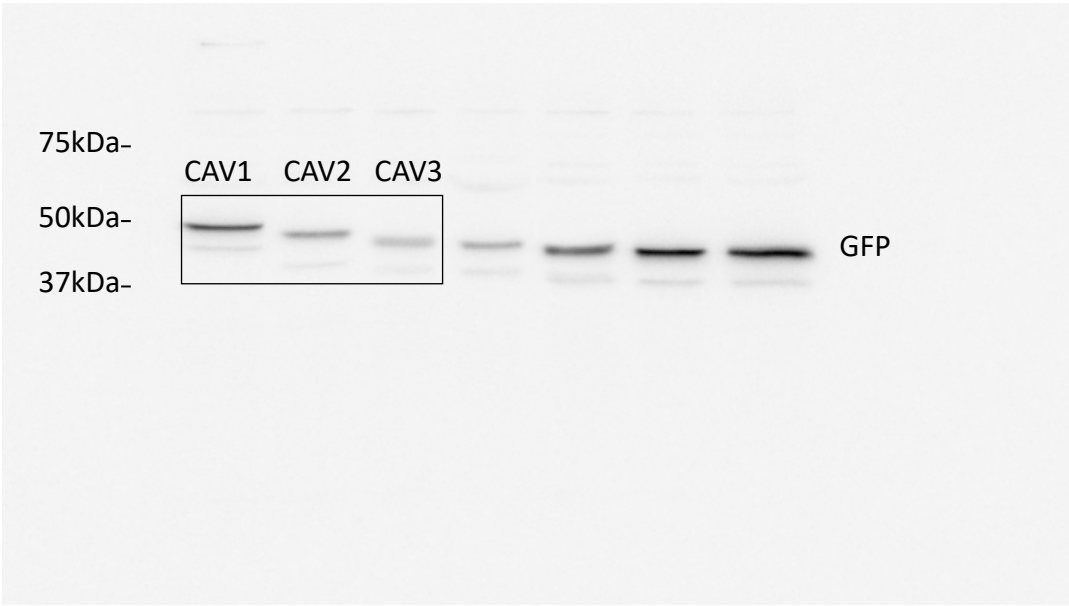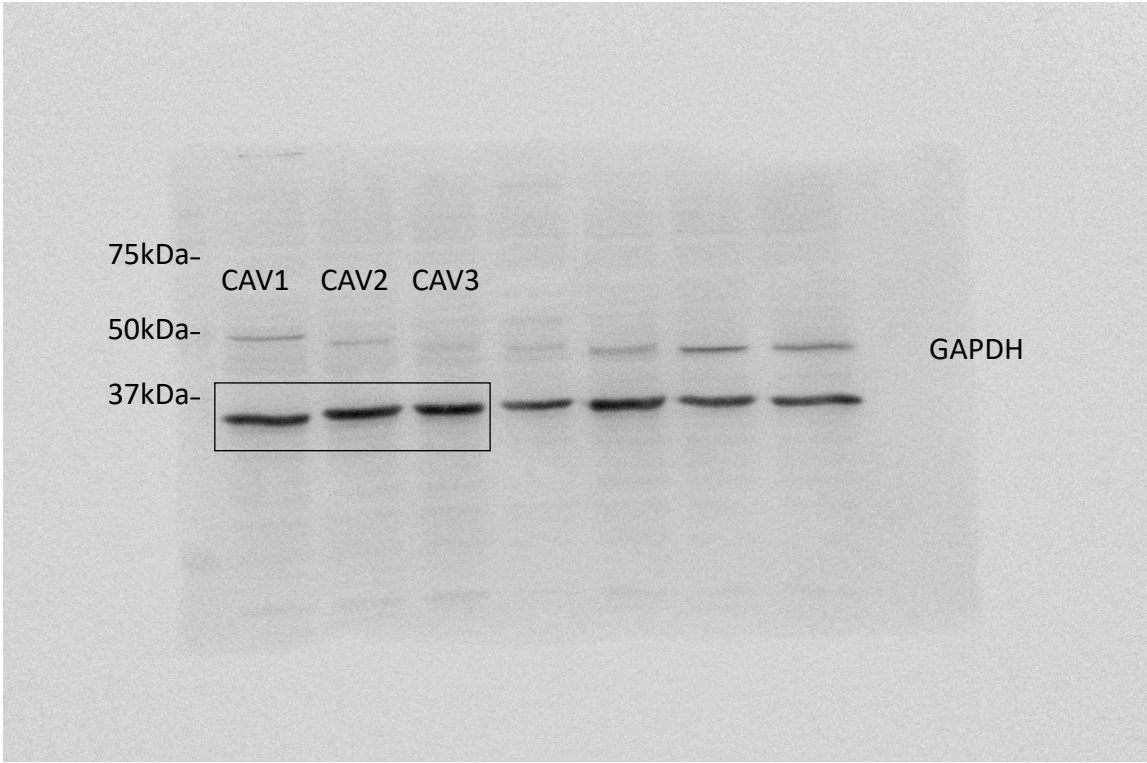

Source Data Fig. 2I

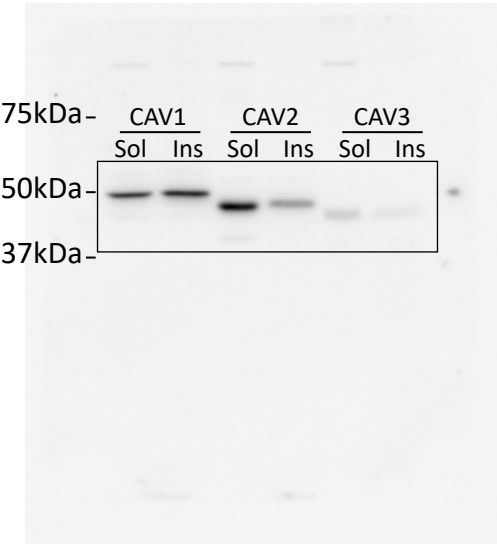

Source Data Fig. 2K

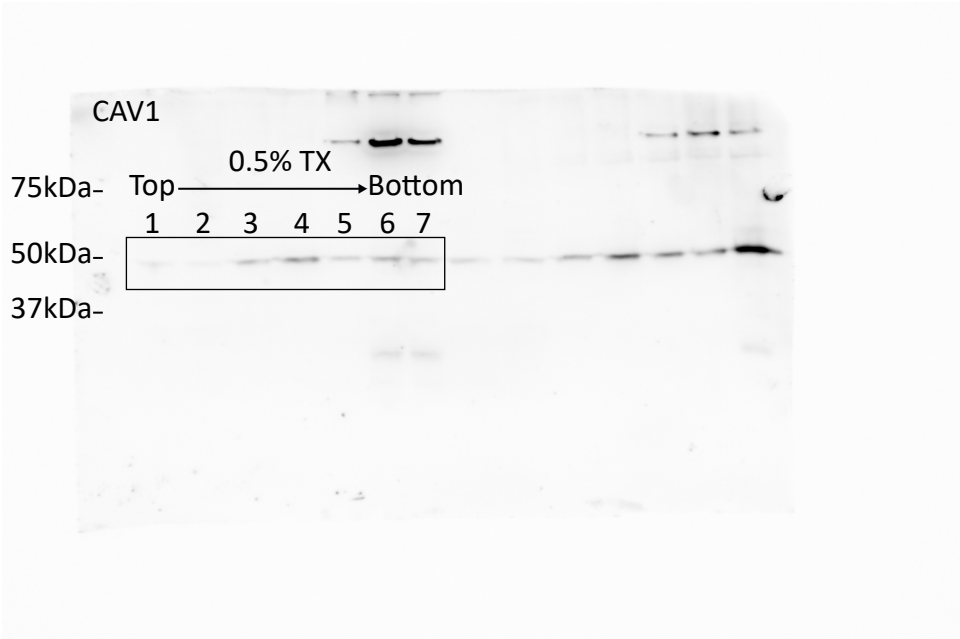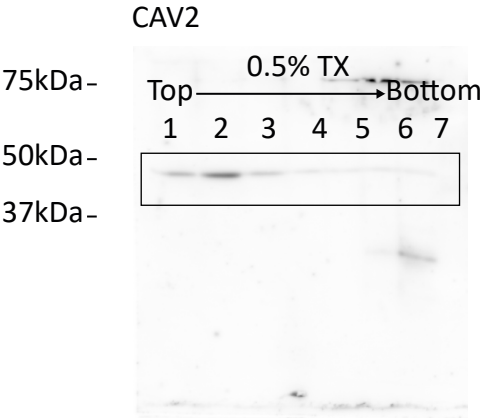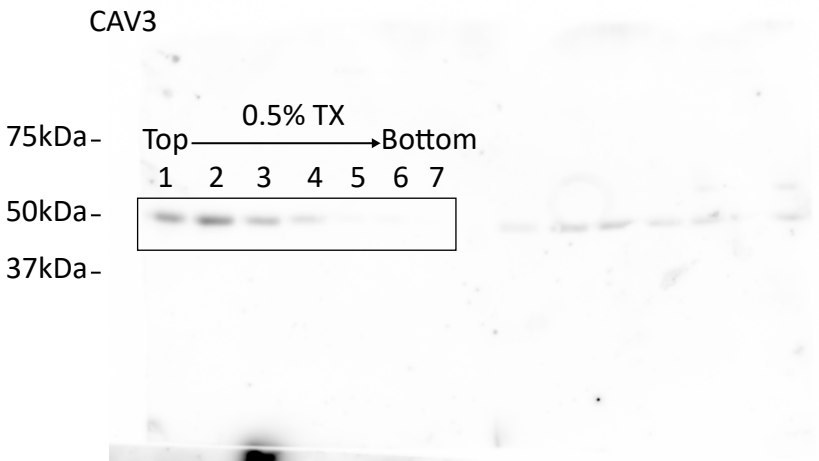

Source Data Fig. 2M

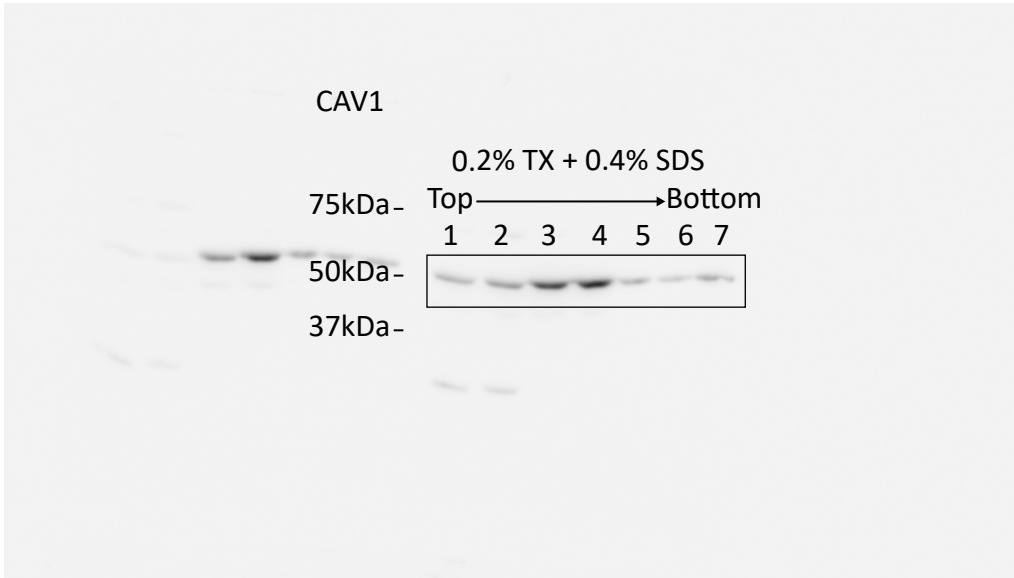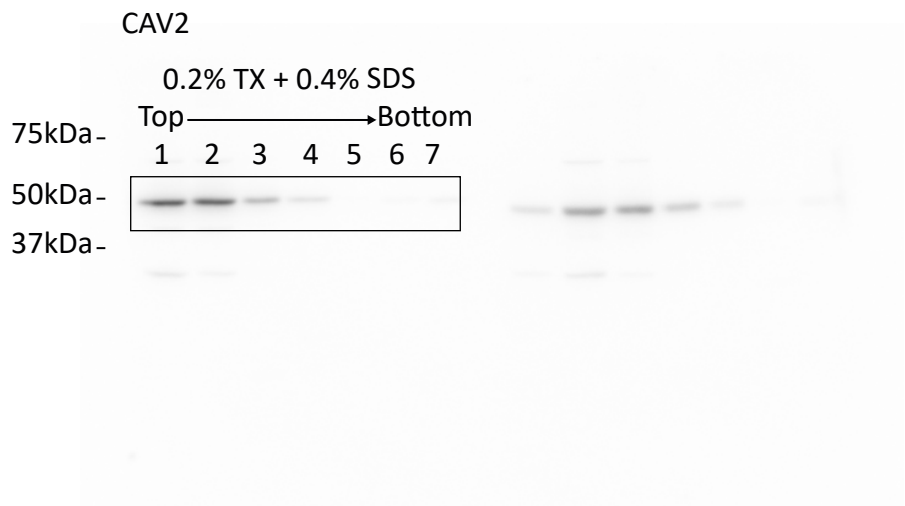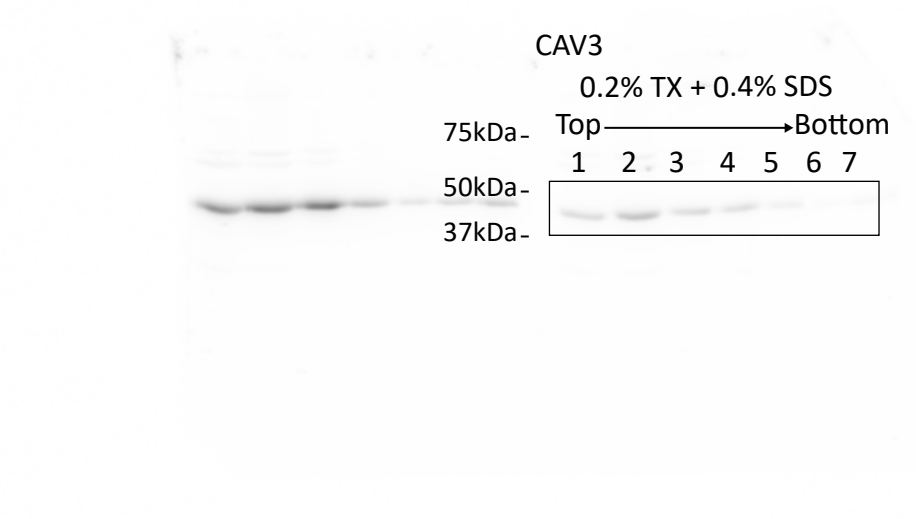

## Source Data Fig. 2Q

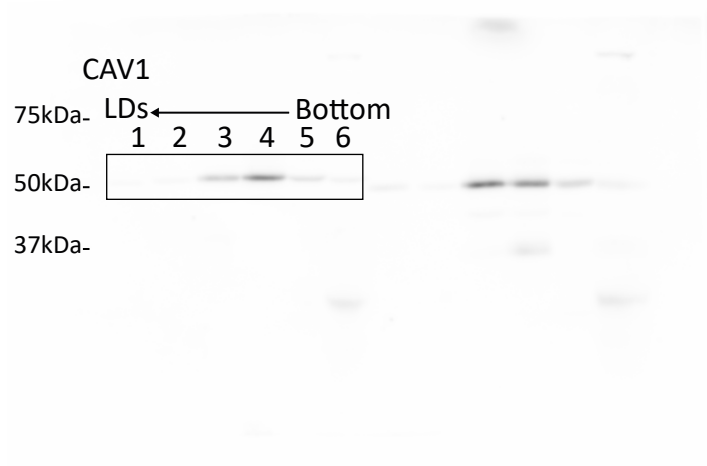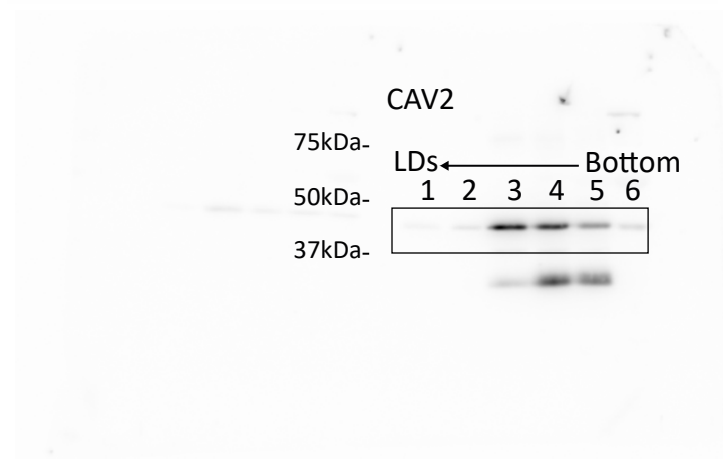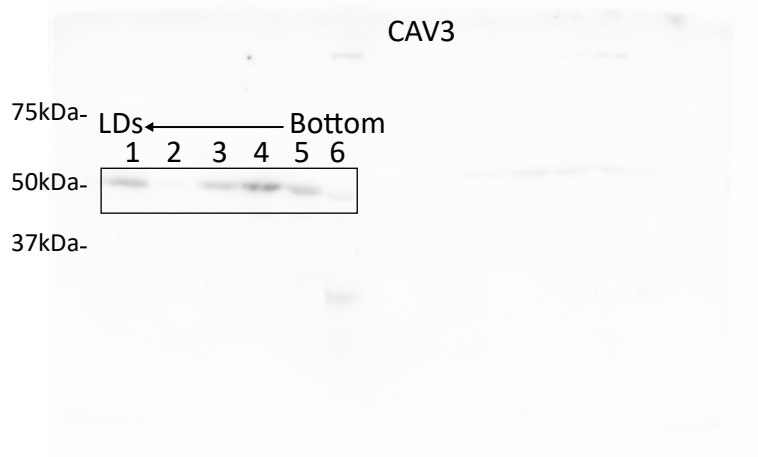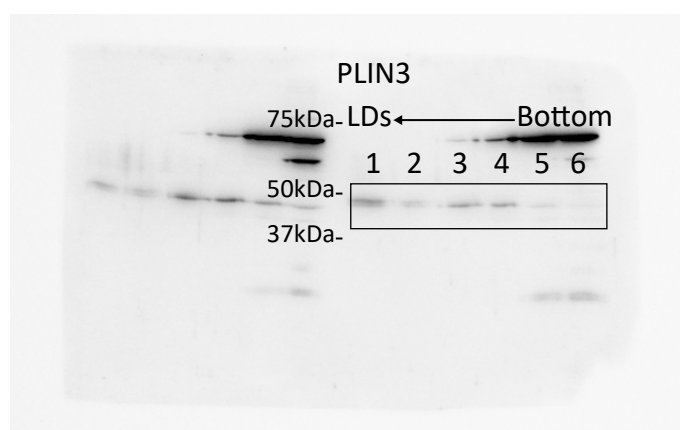

Supplement: SourceData F2 — is the source file for Fig. 2. [file JCB_202204020_SourceDataF2.pdf]

## Source Data Fig. 3A

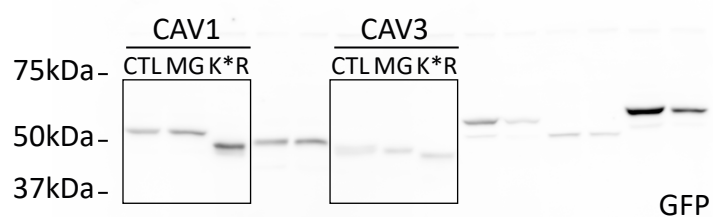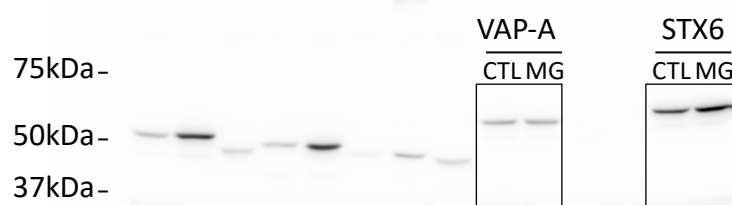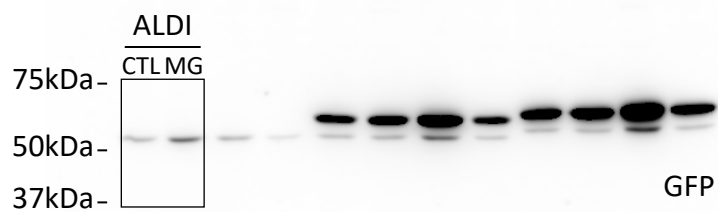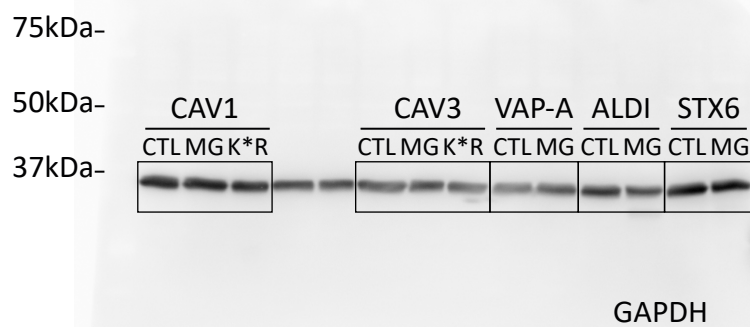

# Source Data Fig. 3H

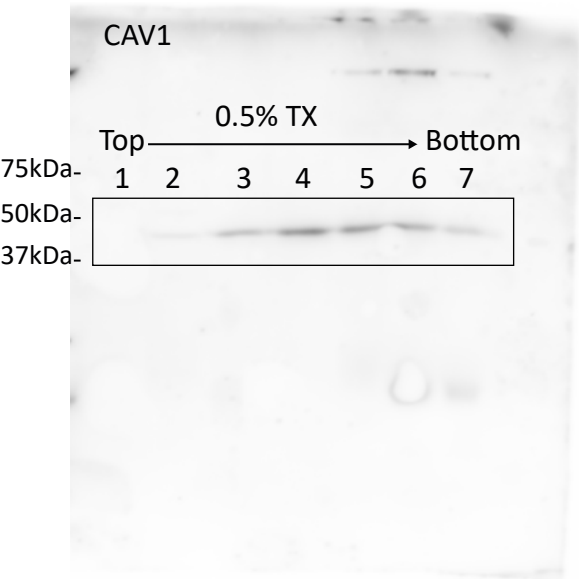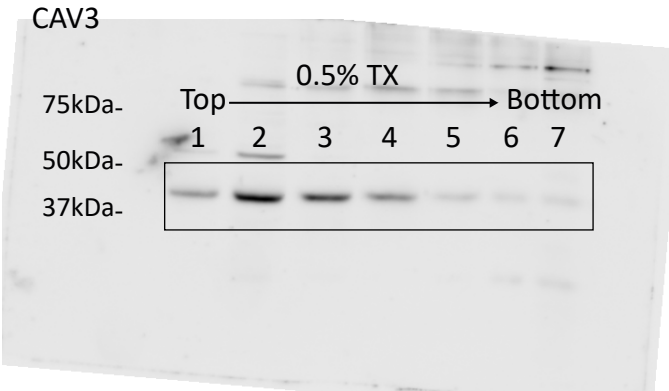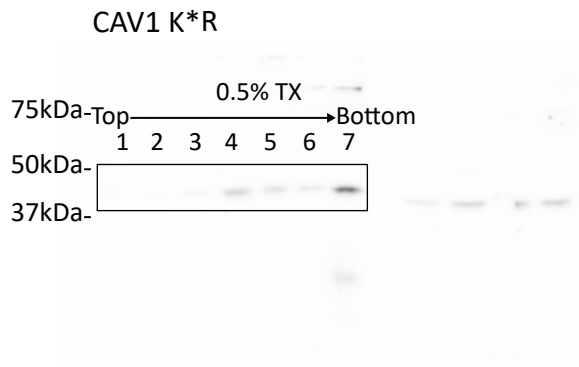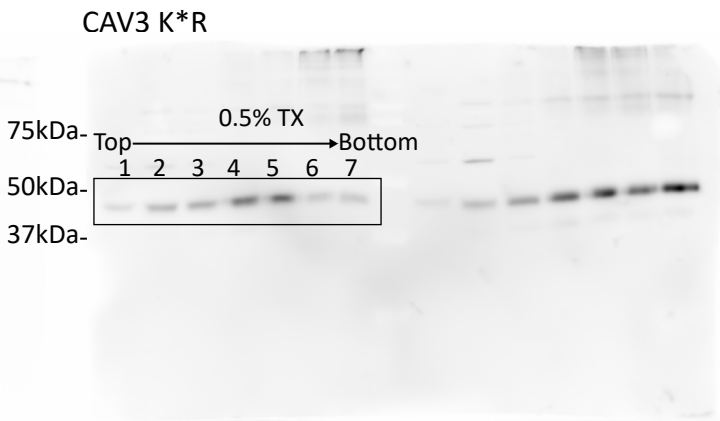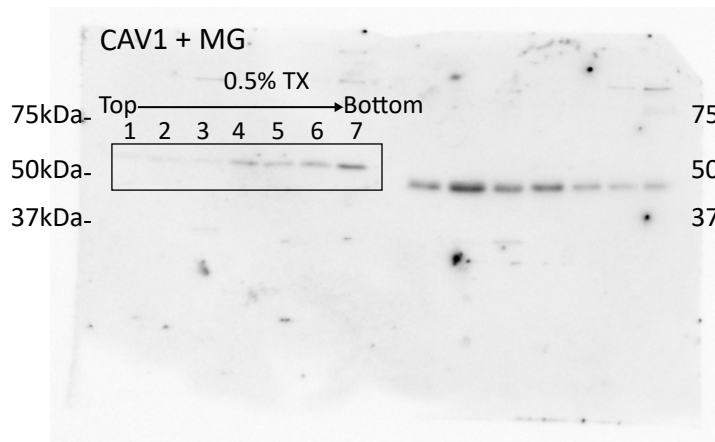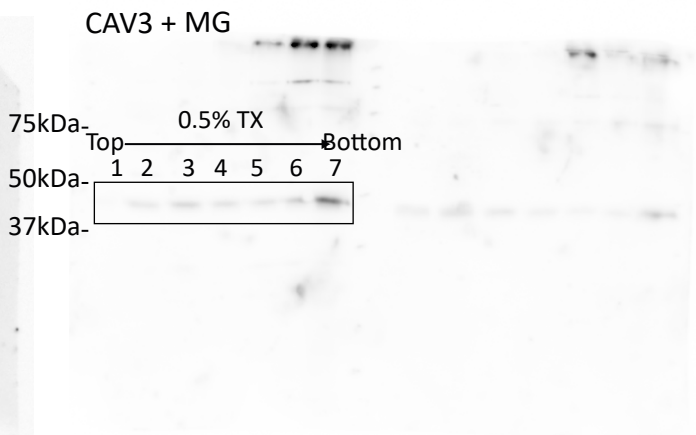

# Source Data Fig. 3K

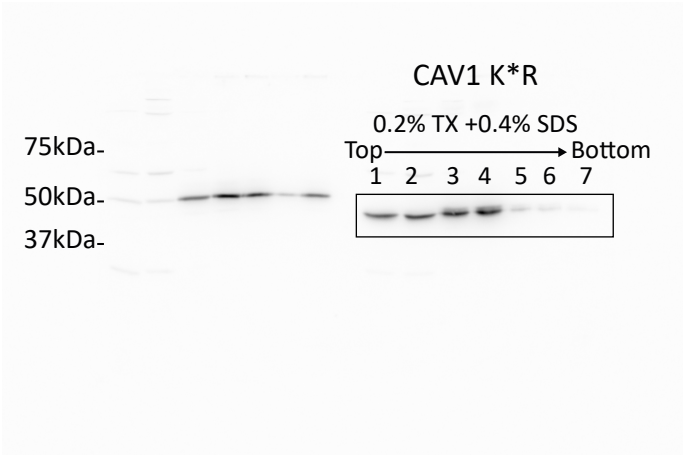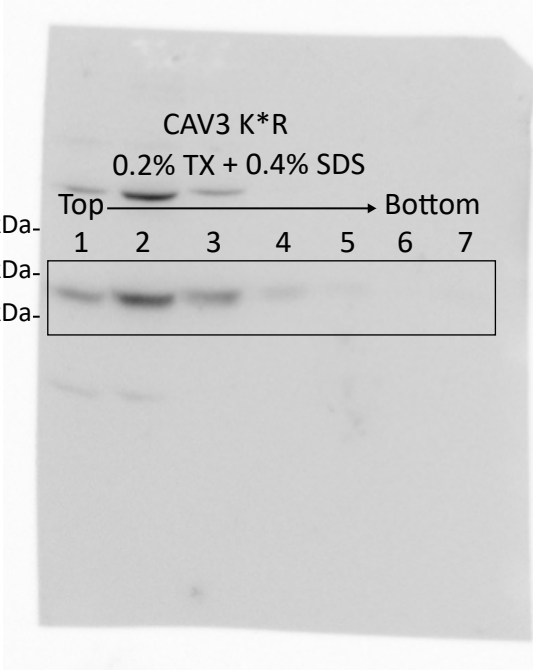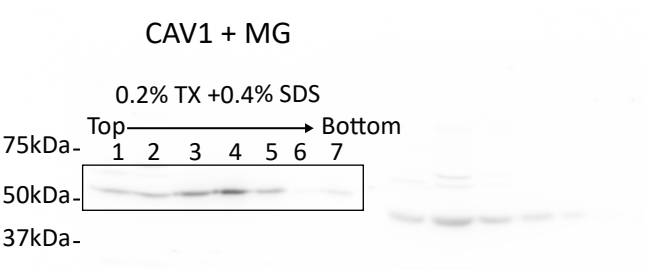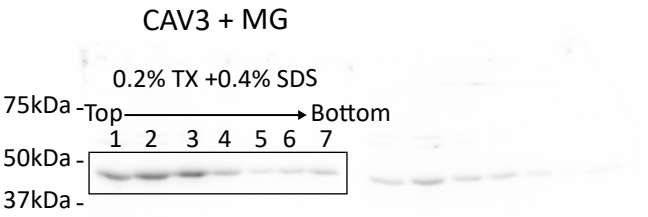

Supplement: SourceData F3 — is the source file for Fig. 3. [file JCB_202204020_SourceDataF3.pdf]

## Source Data Fig. 4A

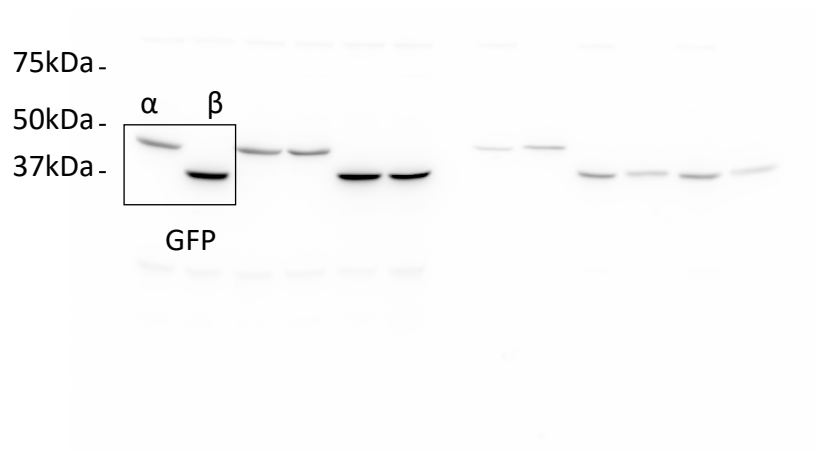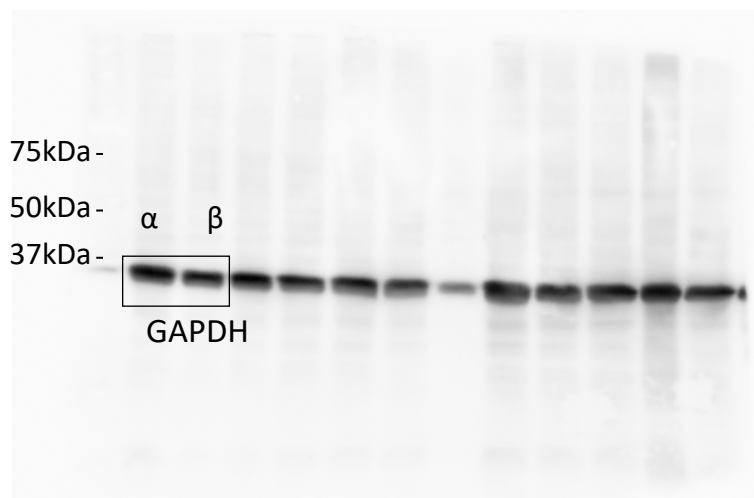

Source Data Fig. 4C

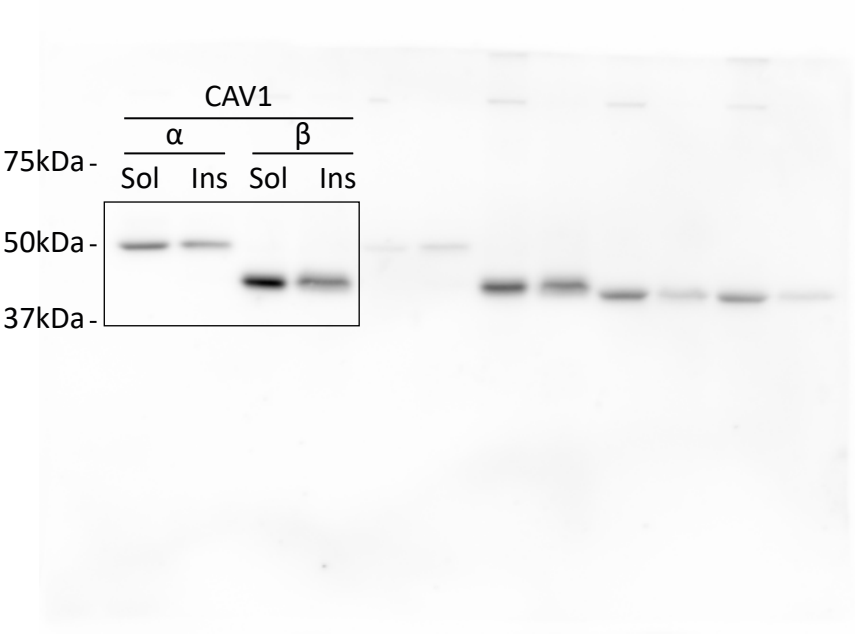

## Source Data Fig. 4I

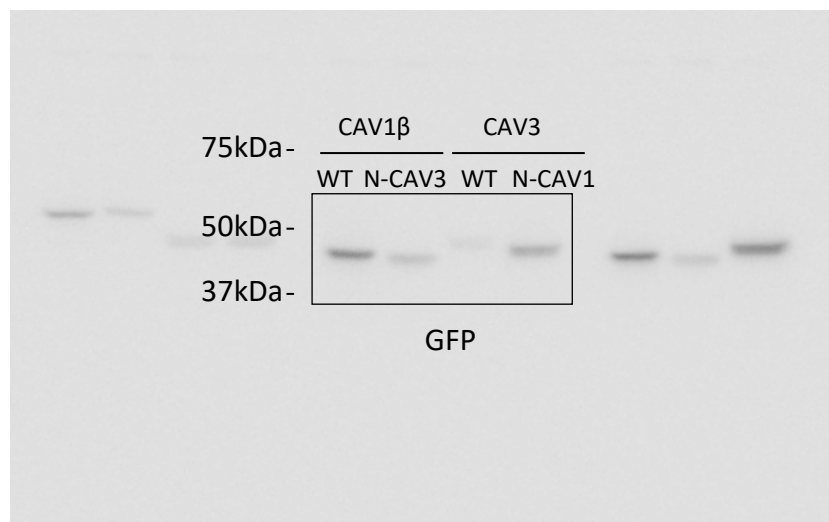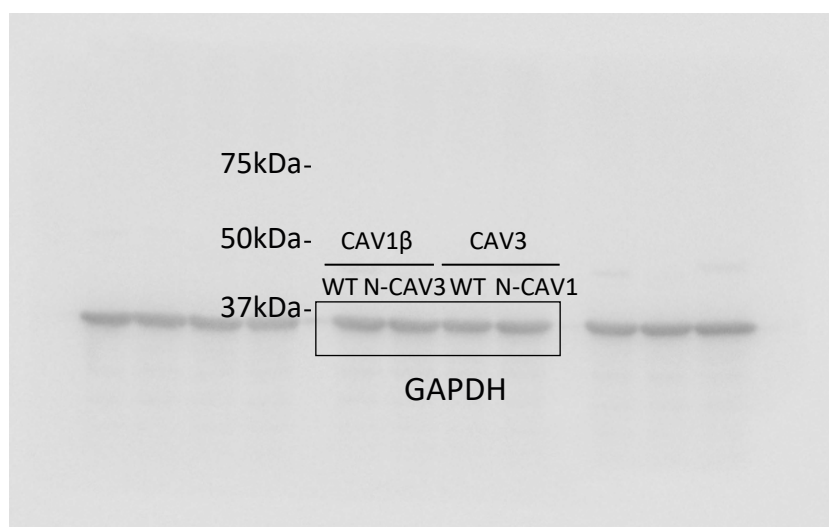

### Source Data Fig. 4J

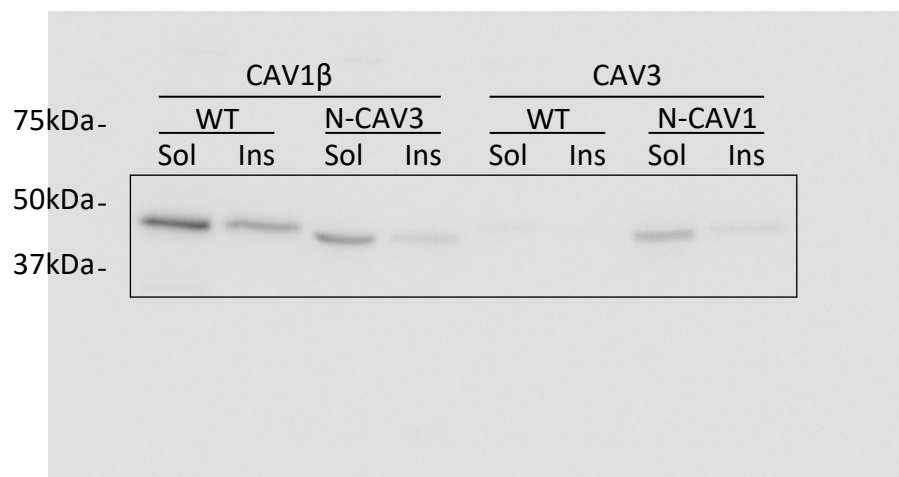

Supplement: SourceData F4 — is the source file for Fig. 4. [file JCB_202204020_SourceDataF4.pdf]

## Source Data Fig. 5B

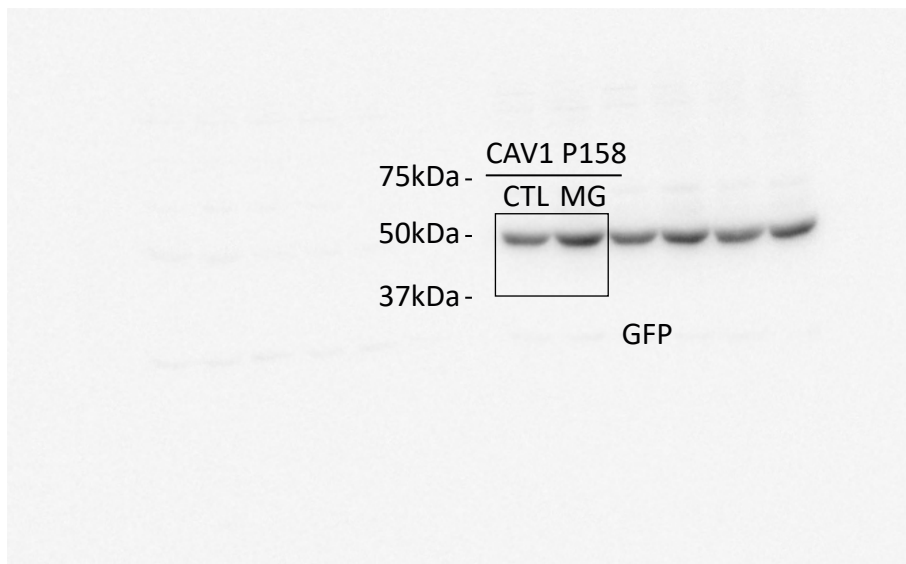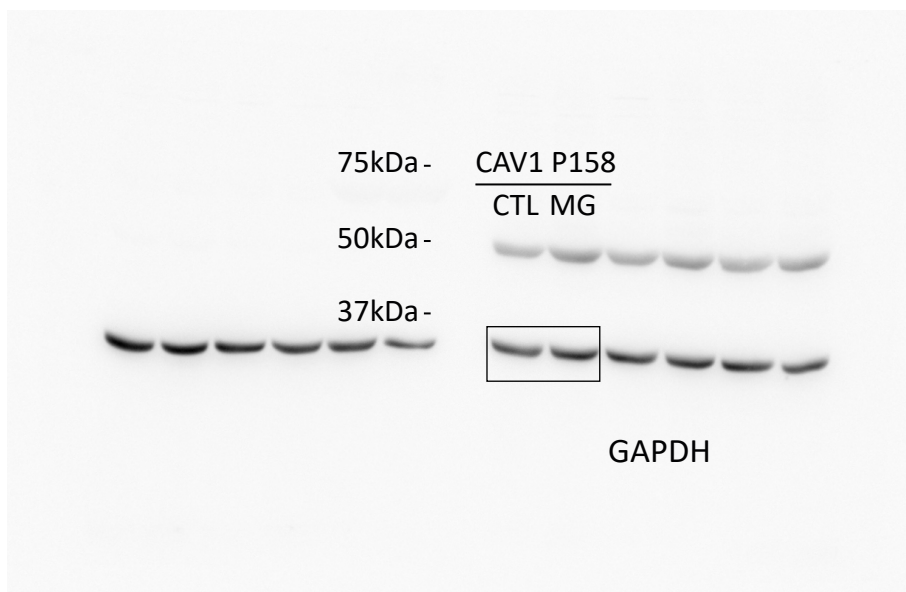

## Source Data Fig. 5E

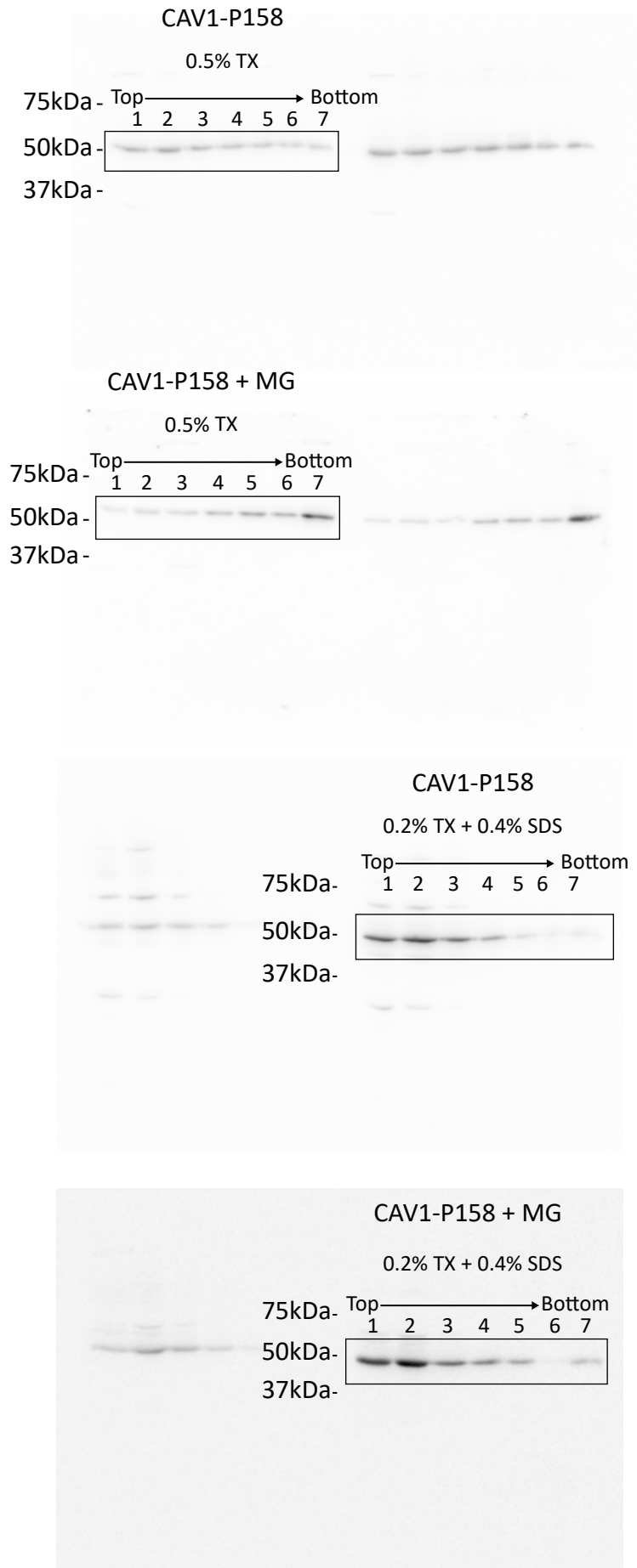

Supplement: SourceData F5 — is the source file for Fig. 5. [file JCB_202204020_SourceDataF5.pdf]

## Source Data Fig. 6B

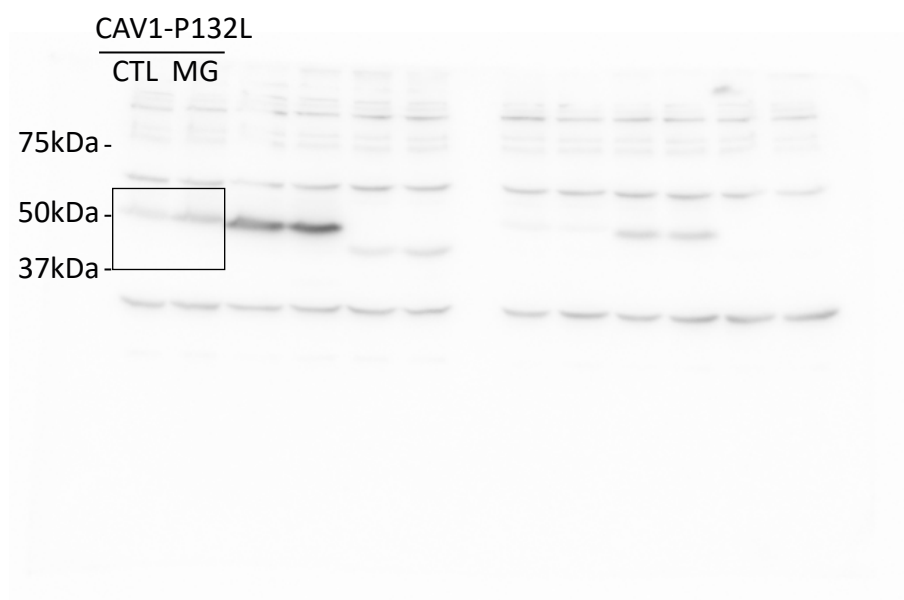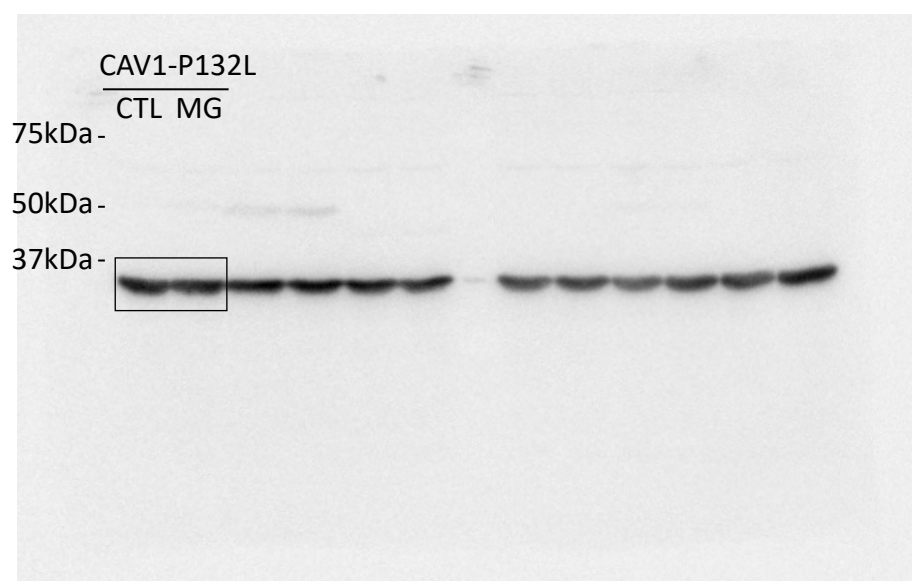

Source Data Fig. 6C

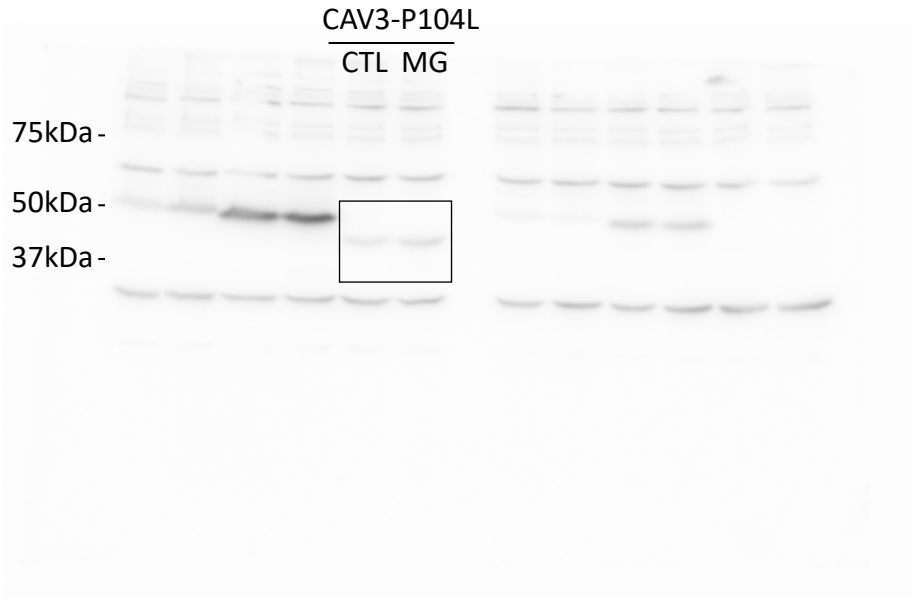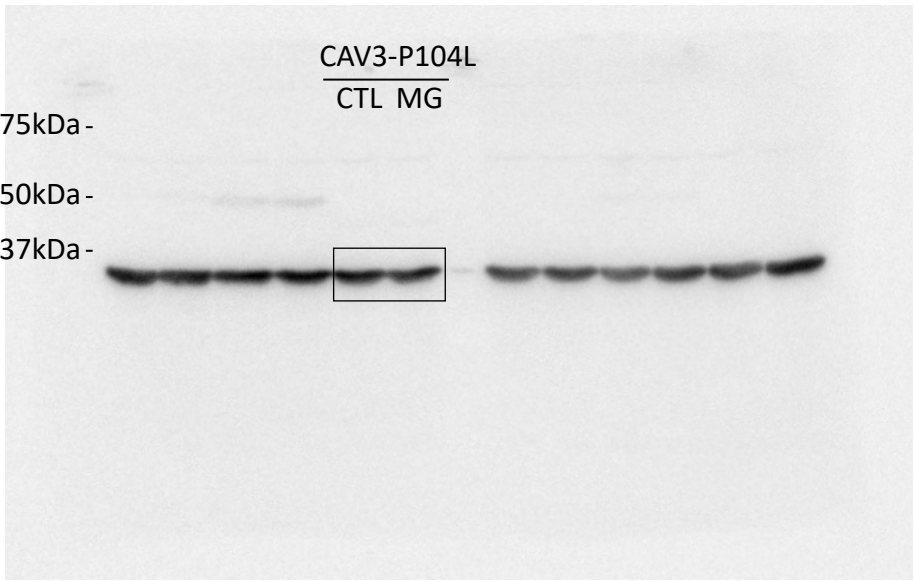

## Source Data Fig. 6H

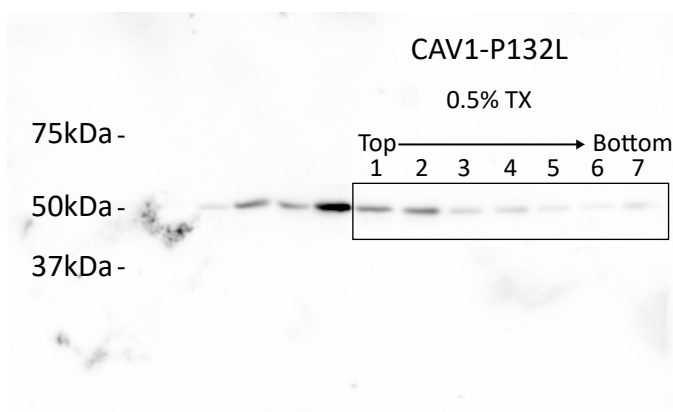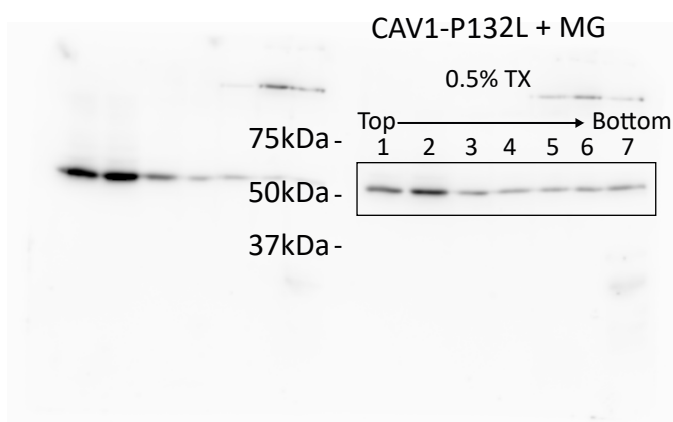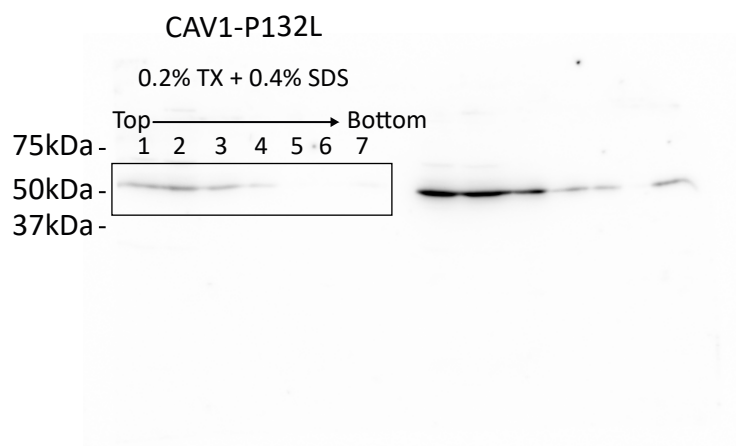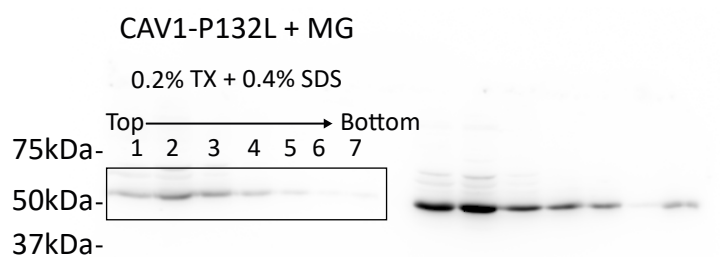

## Source Data Fig.6L

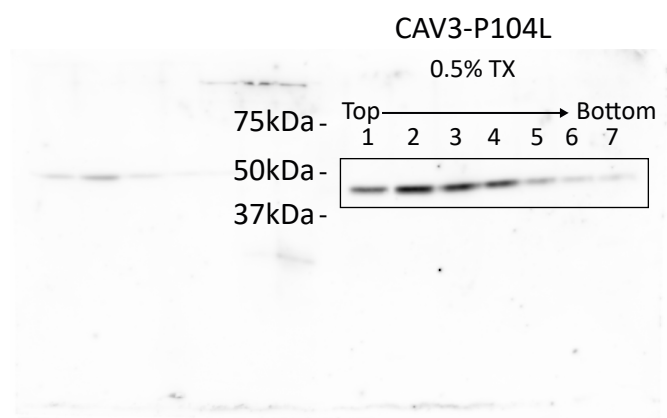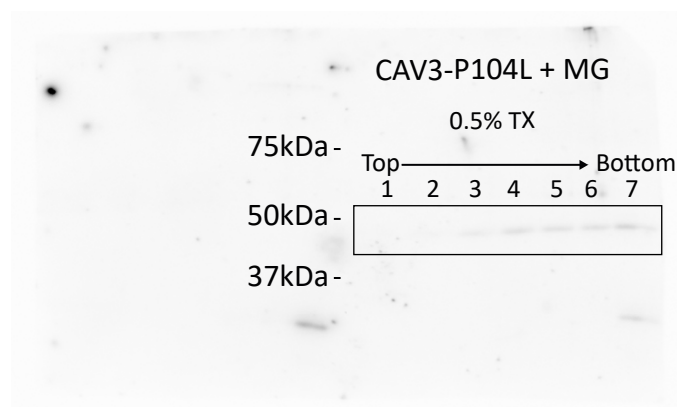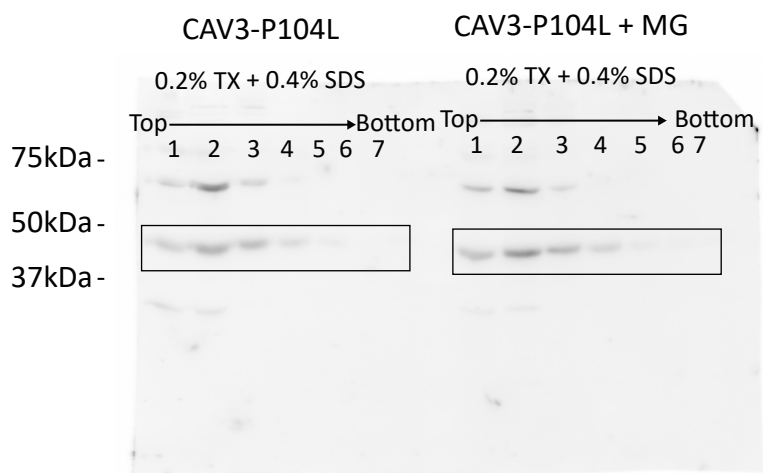

Supplement: SourceData F6 — is the source file for Fig. 6. [file JCB_202204020_SourceDataF6.pdf]

Source Data Fig. S2J

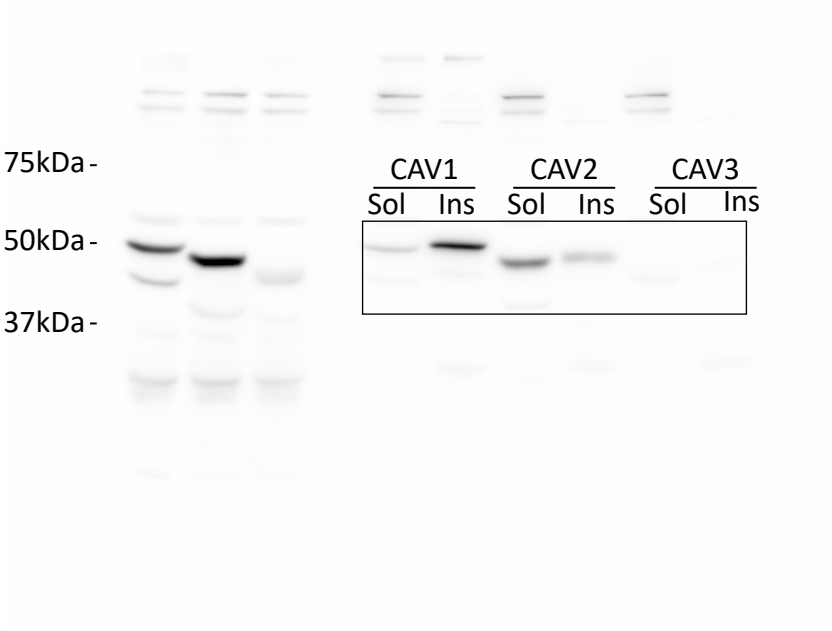

Supplement: SourceData FS2 — is the source file for Fig. S2. [file JCB_202204020_SourceDataFS2.pdf]

## Source Data Fig. S5D

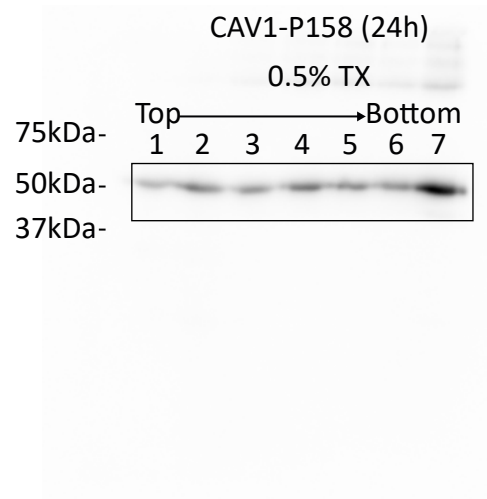

## Source Data Fig. S5F

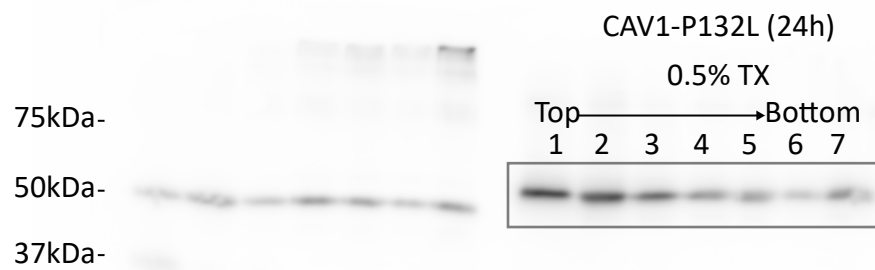

## Source Data Fig. S5H

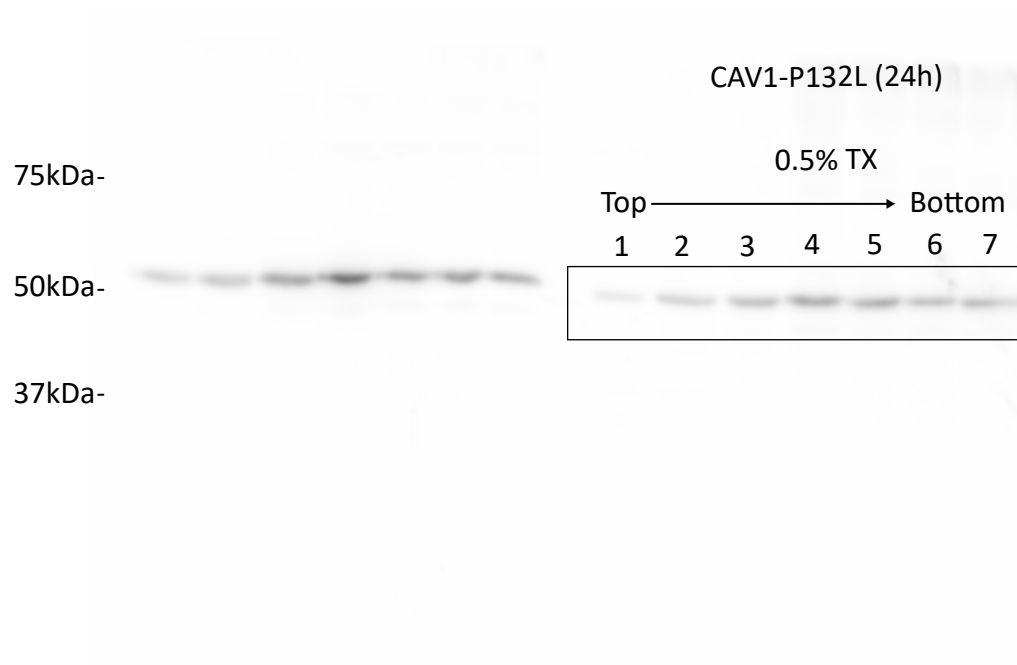

Supplement: SourceData FS5 — is the source file for Fig. S5. [file JCB_202204020_SourceDataFS5.pdf]
